# Supplementary material for: Non-competitive resource exploitation within mosquito shapes within-host malaria infectivity and virulence
Source: Nat Commun. 2018 Aug 27;9:3474. doi: 10.1038/s41467-018-05893-z (PMC6110728; doi:10.1038/s41467-018-05893-z)
Supplement: Supplementary file 1 — Supplementary Information [file 41467_2018_5893_MOESM1_ESM.pdf]

<u>Supplementary Information</u>

Non-competitive resource exploitation within-mosquito shapes within-host malaria infectivity and virulence

Costa *et al.*

SUPPLEMENTARY FIGURES

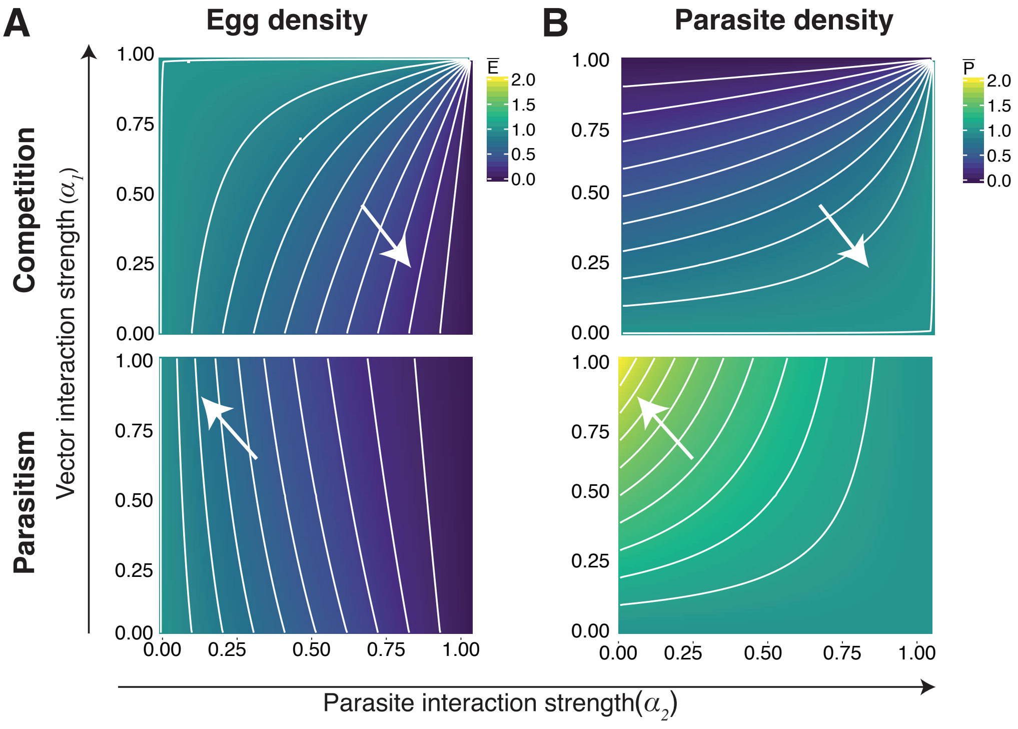

Supplementary Figure 1. Within-vector parasite and egg equilibrium density

Egg (**A**) and parasite (**B**) equilibrium densities for varying values of their interaction strengths (i.e., $\alpha_1$and α_2) assuming both a competitive (upper row) and a parasitic (lower row) scenario. The white arrows point to the egg and parasite density corresponding with the highest value of R_0 shown in Figure 1. Color gradient shows the equilibrium densities for $\bar{E}$ and $\bar{P}$ from highest (lime green) to lowest (dark purple).

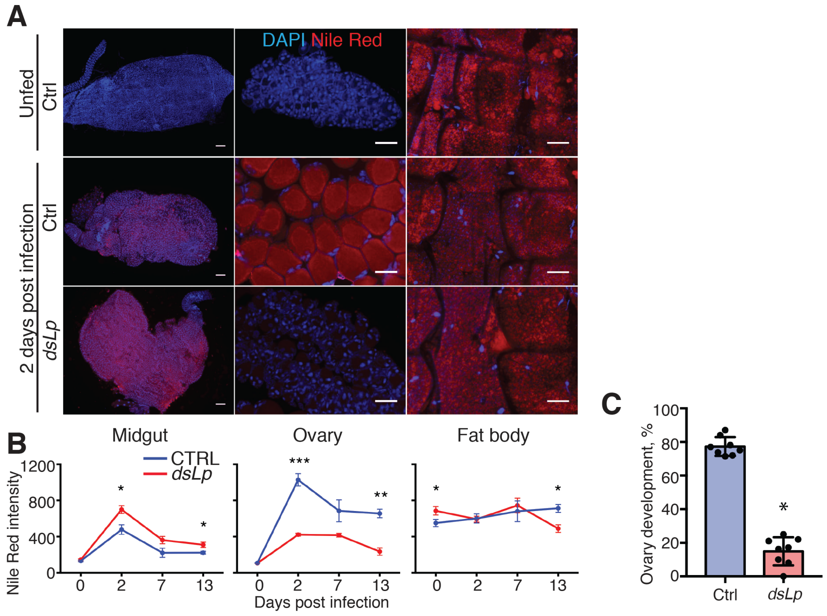

Supplementary Figure 2. Neutral lipid trafficking and ovary development in Lp-depleted mosquitoes.

Distribution of neutral lipids (Nile Red staining) in the midgut, ovaries, and fat body of control (Ctrl) and Lp-deficient (*dsLp*) mosquitoes. (**A**) Representative fluorescence micrographs of female abdomens of control and Lp-depleted mosquitoes dissected before (unfed) and 2 days post *Plasmodium* infection (dpi) (N=3). Neutral lipids are stained by Nile Red (red) and cell nuclei - by DAPI (blue). Scale bar - 100 μm. (**B**) Quantification of the Nile Red staining in the midgut, ovaries, and fat body of control and Lp-depleted mosquitoes before (0) and at 2, 7 and 13 dpi (4-10 mosquitoes per condition per experiment). Each data point represents mean intensity of Nile Red signal ± SEM from pooled experiments (N=3). *: $p<0.05$, **: $p<0.001$, ***: $p<0.0001$; two-sided Mann-Whitney test comparing Ctrl and *dsLp* at each time point. (**C**) Proportion of control (Ctrl) and Lp-deficient (*dsLp*) mosquitoes with developed ovaries (19-88 mosquitoes per condition per experiment, N=8). Ovary development was gauged 18/19 dpi and ovaries containing 3 fully grown eggs or more were scored as positive (Rono et al., 2010). Each dot shows the percentage of developed ovaries per experiment and the bar plot shows mean and SD. *: $p<0.05$, one-sided

Wilcoxon matched-pairs signed rank test, paired values for *dsRNA* treatment per experiment.

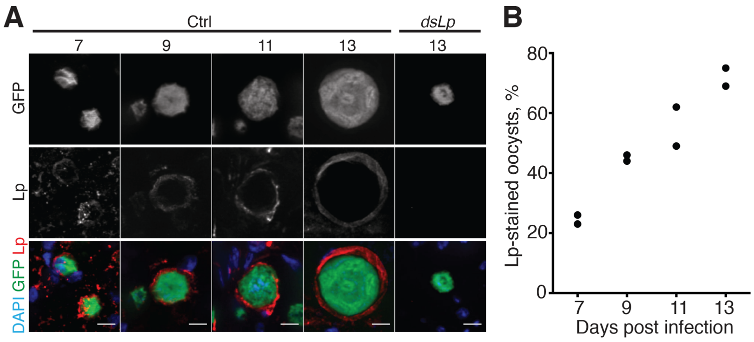

Supplementary Figure 3. Lp accumulation around *Plasmodium* oocysts.

Lp uptake of *P. berghei* oocysts was quantified by fluorescence microscopy in control (Ctrl) and Lp-deficient (*dsLp*) mosquitoes. (**A**) Lp accumulation (red) on the GFP-expressing oocysts (green) revealed by anti-Lp antibodies at 7, 9, 11, and 13 days post infection (dpi). No Lp signal was observed in Lp-depleted mosquitoes. Representative pictures from 2 independent experiments are shown. Nuclei are visualized by DAPI (blue). Scale bars - 10 µm. (**B**) Dot plot showing quantification of Lp signal (N=2). Each dot shows the percentage of Lp-stained oocysts per experiment (45-562 oocysts per time point per experiment). Between 6 and 13 mosquitoes were analyzed per condition per experiment, N=2.

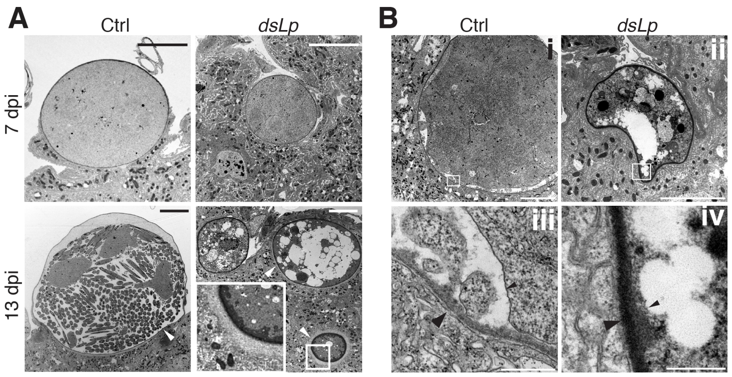

Supplementary Figure 4. Effect of Lp depletion on oocyst ultrastructural morphology.

(**A**) Representative transmission electron micrographs of *P. berghei* oocysts in control (Ctrl) and Lp-depleted (*dsLp*) mosquitoes 7 (N=1) and 13 (N=3) days post infection (dpi) (5-14 mosquitoes per condition per experiment). Oocysts 13 dpi in Lp-depleted mosquitoes displayed abnormal cytoplasmic vacuolization and failed to sporulate as control oocysts. In control mosquitoes, white arrowheads point to the membranous structures, whereas in Lp-depleted mosquitoes they show the electron-dense actin-zone associated with dead parasites (Shiao et al., 2006). Scale bars - 10 µm. **(B)** Representative transmission electron micrographs of *P. berghei* oocysts 13 dpi in control (**i**) and Lp-depleted (**ii**) mosquitoes. Scale bars - 5 µm. (**iii**) Detailed images of the membranous structures showing normal membrane retraction in control oocysts. (**iv**) Detailed image of electron dense membranous structures and of abnormal cytoplasmic vacuolization in Lp-depleted oocysts. Scale bars - 0.5 µm.

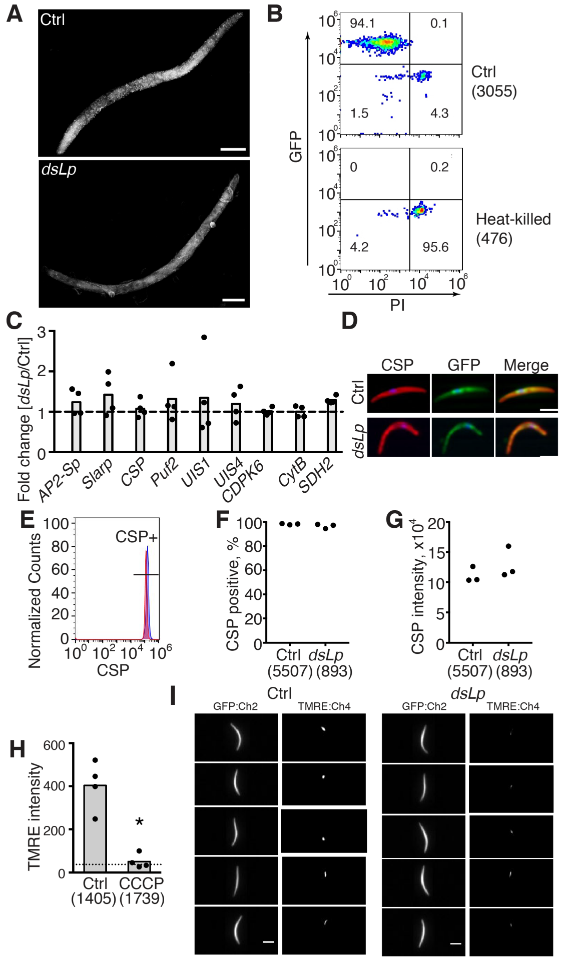

Supplementary Figure 5. Morphological, transcriptional and metabolic characterization of *P. berghei* sporozoites isolated from control and Lp-depleted mosquitoes.

(A) Representative scanning electron micrographs of *P. berghei* salivary gland sporozoites isolated at 18 days post infection (dpi) from control (Ctrl) and Lp-depleted (*dsLp*) mosquitoes (7-13 mosquitoes per condition). Scale bars - 1 µm. Salivary gland sporozoites were labelled with a mouse monoclonal antibody against CSP and secondary gold-conjugated antibody. **(B)** Sporozoite propidium iodide (PI) staining and endogenous GFP fluorescence were measured by imaging flow cytometry (22-24 mosquitoes per condition per experiment). Fluorescence intensity dot plots from one representative experiment (N=3) show PI and GFP signals in control (top panel) and heat-killed (lower panel) sporozoites. The total numbers of sporozoites are

shown in brackets. (**C**) Fold changes in transcript levels of sporozoite developmental (*AP2-Sp*, *Slarp*, *CSP*, *Puf2*, *UIS1*, *UIS4* and *CDPK6*) and mitochondrial (*CytB*, *SDH2*) genes were analyzed by quantitative RT-PCR (qRT-PCR) in the salivary gland sporozoites isolated at 18 dpi from control and Lp-depleted mosquitoes (28-63 mosquitoes per condition per experiment, N=4). Relative expression levels of each gene were normalized to the expression levels of the *P. berghei* ribosomal RNA gene *18s* and expressed as fold-change relative to controls (dashed line). Each dot shows fold-change in gene expression per experiment and bars show the mean fold change. **(D)** Surface distribution of CSP revealed by a mouse monoclonal antibody on the GFP-expressing *P. berghei* salivary gland sporozoites isolated 18 dpi from control (Ctrl) and Lp-depleted (*dsLp*) mosquitoes. Nuclei are visualized by DAPI (blue). Scale bars - 5 µm. (**E-G**) CSP protein levels on the surface of *P. berghei* sporozoites isolated from control (Ctrl, blue) and Lp-depleted (*dsLp,* red) mosquitoes were quantified by imaging flow cytometry (12-35 mosquitoes per condition per experiment, N=3). Fluorescence intensity histograms from one representative experiment (**E**), proportions of CSP-positive sporozoites (**F**) and CSP geometric MFI (**G**) of each experimental replicate are shown. Total numbers of analyzed sporozoites are shown in brackets. Statistical significance of the results was evaluated by one-sided paired *t*-test, paired values for *dsRNA* treatment per experiment p=0.0823 (F); p=0.2194 (G). (**H**) The mitochondrial membrane potential was measured by imaging flow cytometry of TMRE stained sporozoites isolated from 28-88 mosquitoes per condition per experiment. Dashed line shows MFI of unstained control sporozoites as compared to sporozoites stained with TMRE before (Ctrl) or after treatment with the mitochondrial potential decoupling drug CCCP. Each dot represents the geometric MFI of TMRE in each experiment (N=4) and the bar plot indicates the mean. The total numbers of analyzed sporozoites are shown in brackets. *: $p<0.05$, one-sided paired *t*-test, paired values for drug treatment per experiment. (**I**) Representative images (Figure 4C, one experiment) of control (Ctrl) and lipid-deprived (*dsLp*)

sporozoites stained with TMRE (42-49 mosquitoes per condition per experiment). Scale bars - 5 µm.

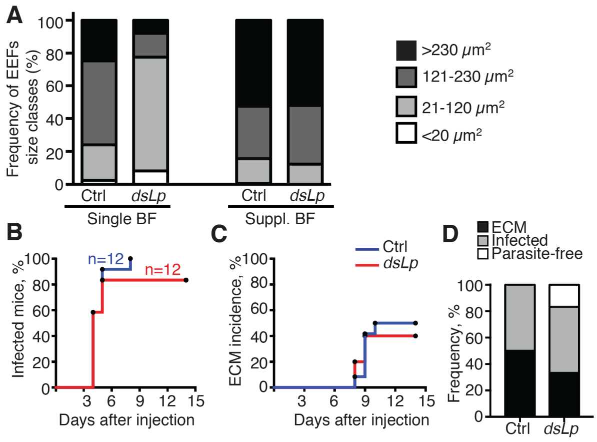

Supplementary Figure 6. Supplemental feeding restores infectivity and virulence of lipid-deprived sporozoites *in vitro* and *in vivo*.

(**A**) Human hepatoma HepG2 cells were infected with 10,000 *P. berghei* sporozoites isolated from control (Ctrl) and Lp-deficient (*dsLp*) mosquitoes without (Single BF) or after a supplemental blood feeding 7 days post infection (dpi) (Suppl. BF). Samples from Figure 4. Four EEF classes are indicated by color gradient, ranging from white for abortive EEFs with smallest size (<20 μm^2) to black for mature EEFs with largest size (>230 μm^2). The bar plots display mean frequencies of pooled experiments (N=3). (**B**) Kaplan-Meier analysis of time to blood stage parasitemia, (**C**) incidence (%) of experimental cerebral malaria (ECM), and (**D**) cumulative health status of mice infected by subcutaneous injection of 5,000 sporozoites dissected from the salivary glands of control (Ctrl) or Lp-depleted (*dsLp*) mosquitoes after a supplemental blood meal 7dpi (N=2, total number of mice in both conditions n=12; p=0.4444 (B) and p=0.8026 (C); log-rank test).

A

$$R_0 = \frac{\beta_0 \alpha_2 \bar{P}}{\delta_v + \nu_v + \gamma_v} S_v \frac{\beta_0 \bar{P}}{\delta_h + \nu_0 \alpha_2 \bar{P}/(h + \bar{P}) + \gamma_h} S_h$$

B

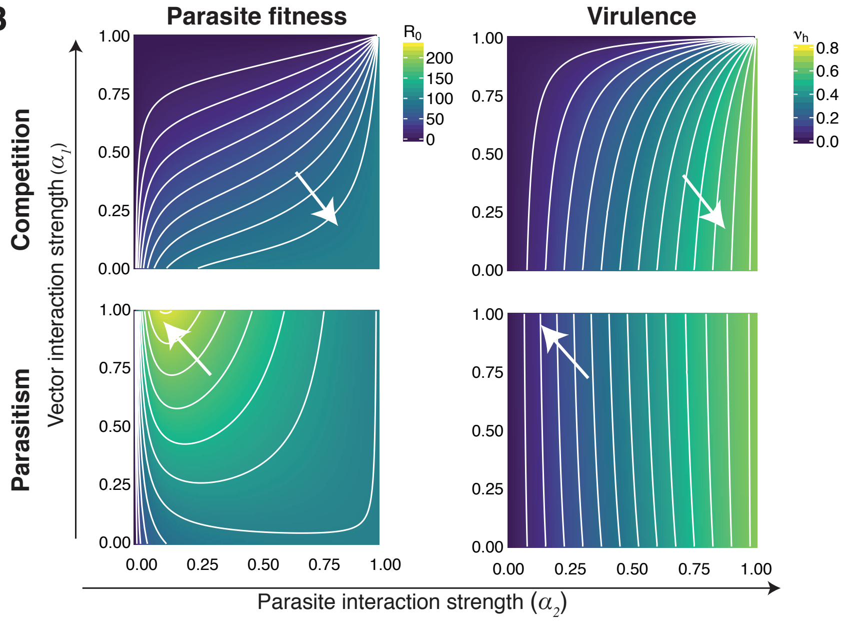

Supplementary Figure 7. Parasite fitness and virulence assuming non-linear virulence relationships.

(A) Expression of the parasite fitness (R_0) assuming a non-linear relationship in the human virulence ν_h. For this model, the parasite fitness R_0 and the virulence in the human host ν_h **(B)** are shown for various values of α_1 and α_2 assuming both a competitive (upper row) and a parasitic (lower row) scenario. The white arrows point to the contours with the highest value of R_0 and their corresponding virulence values ν_h. Note that, similar to the linear model, there is a maximum in R_0 only in the parasitic scenario (lime green color in the heatmap), occurring at low virulence values. The parameter values used in this model are fully described in Supplementary Table 1.

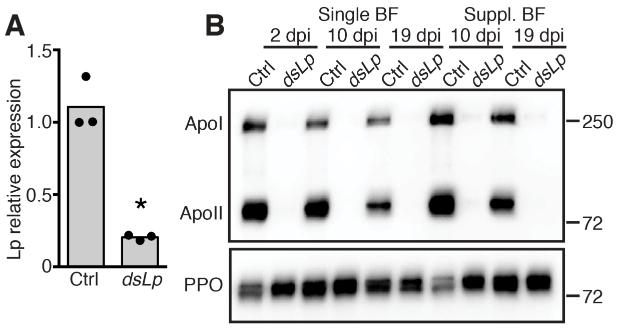

Supplementary Figure 8. Efficiency of *Lp* knockdown.

(**A**) *Lp* transcript levels were measured by quantitative RT-PCR (qRT-PCR) in *dsLp*- and *dsLacZ*-injected mosquitoes (*7b*) before feeding (15 mosquitoes per condition per experiment). Each dot represents levels of *Lp* expression normalized to expression level of the ribosomal gene *RPL19* per experiment and the bar plot shows the mean (N=3, *: $p<0.05$; one-sided paired *t*-test, paired values for *dsRNA* treatment per experiment). (**B**) Immunoblotting analysis of Lp protein levels in the hemolymph from G3 control and Lp-depleted (*dsLp*) mosquitoes (8-15 mosquitoes per condition) using anti-Lp monoclonal antibodies (Rono et al., 2010). Hemolymph was collected 2, 10, and 19 days post *P. berghei* infection (dpi). Levels of the hemolymph-borne mosquito enzyme prophenoloxidase 2 (PPO2) were used as a loading control.

SUPPLEMENTARY TABLES

Supplementary Table 1: Modelling parameter values used in this study.

Parameter	Value	Description
**Within-vector model**		
$\boldsymbol{r_1}$	0.2 day^{-1}	Egg intrinsic growth rate
$\boldsymbol{r_2}$	0.5 day^{-1}	Parasite intrinsic growth rate
$\boldsymbol{K_1}$	1	Egg carrying capacity
$\boldsymbol{K_2}$	1	Parasite carrying capacity
$\boldsymbol{\alpha_1}$	[0,1]	Egg competition coefficient
$\boldsymbol{\alpha_2}$	[0,1]	Parasite competition coefficient
**Epidemiological dynamics**		
$\boldsymbol{S_h}$	10	Susceptible humans
$\boldsymbol{S_v}$	1000	Susceptible vectors
$\boldsymbol{\beta_0}$	0.025	Basic transmission rate of the parasite
$\boldsymbol{\delta_h}$	$1\ x\ e^{-5}$ day^{-1}	Human intrinsic death rate
$\boldsymbol{\delta_v}$	0.03 day^{-1}	Parasite intrinsic death rate
$\boldsymbol{\nu_0}$	1 day^{-1}	Basic parasite virulence in humans
$\boldsymbol{\nu_v}$	0.06 day^{-1}	Parasite virulence in vectors
$\boldsymbol{\gamma_h}$	0.025 day^{-1}	Human recovery rate
$\boldsymbol{\gamma_v}$	0.001 day^{-1}	Parasite recovery rate
$\boldsymbol{h}$	0.5	Parasite density at which virulence is half maximal

Supplementary Table 2: Sample size of the experiments performed in this study.

		Sample size (n)	
**Figure**	**Replicate (N)**	**Ctrl**	***dsLp***
2A-B	1	4	4
	2	2	2
	3	1	1
	4	3	3
	total mice	10	10
2C-F	1	6	5
	2	6	5
	3	6	6
	total mice	18	16
3A-B	1	6	9
	2	3	8
	3	21	8
	total mosquitoes	30	25
3C	1	5 (D13)	6 (D13)
	2	7 (D13)	7 (D13)
	3	8 (D7); 14 (D13)	8 (D7); 10 (D13)
	total mosquitoes	8 (D7); 26 (D13)	8 (D7); 23 (D13)
3D (*Pb*)	1	10	16
	2	6	9
	3	3	8
	4	29	14
	5	6	6
	6	21	8
	total mosquitoes	75	61
3D (*Pf*)	1	31	24
	2	5	21
	3	26	30
	total mosquitoes	62	75
3E *(Pb)*	1	55	25
	2	10	9
	3	19	22
	4	26	23
	5	43	29
	6	31	39
	total mosquitoes	184	147
3E (*Pf*)	1	4	3
	2	14	5
	3	47	75
	4	27	50
	5	33	32
	6	22	52
	total mosquitoes	147	217
3F	1	12	12
	2	24	22

	3	30	35
	total mosquitoes	66	69
3G-H	1	53	88
		Sample size (n)	
**Figure**	**Replicate (N)**	**Ctrl**	***dsLp***
	2	60	58
	3	42	49
	4	28	38
	total mosquitoes	183	233
4A (ooc)	1	9 (D7), 8 (D9), 5	14 (D7), 7 (D9), 12
	2	6 (D7), 8 (D9), 7 (D11), 8 (D13)	7 (D7), 8 (D9), 7 (D11), 9 (D13)
	total mosquitoes	15 (D7), 16 (D9),	21 (D7), 15 (D9),
4A (eggs)	1	7 (D0), 7 (D2), 5	10 (D0), 11 (D2),
	2	5 (D0), 5 (D2)	8 (D0), 7 (D2)
	3	6 (D0), 8 (D2)	5 (D0), 7 (D2)
	total mosquitoes	18 (D0), 20 (D2), 5	23 (D0), 25 (D2),
Bi	1	54	52
	2	46	48
	3	33	59
	4	63	38
	5	28	38
	total mosquitoes	224	235
Bv	1	13	20
	2	46	33
	3	16	3
	3	55	28
	4	26	36
	total mosquitoes	156	120
Bii-iv	1	3	2
	2	3	3
	3	3	1
	total wells	9	6
Bvi-vii	1	3	3
	2	3	3
	3	3	3
	total wells	9	9

Supplementary Table 3: Gene fragments used to generate *dsRNA* for RNAi in this study.

Gene	Sequence (5' – 3')
Lp	AGCGATCCGGCCATGGCTTGAGTTCGTGAAGGCTGCCACCAAGATCTTCCAGCAGGTGAGCGAAGTGTTCGG<br>CCAGCTGTCGCAGGTGTACGTGGAAAGCTTCCGCAAGATGTCCGCCCTGGTGAACGACGTCGTCGCCCAGC<br>TGATGGAAACGTTCAACACGAAGGTACTGCCGGCGCTGAAGGAGCTGTCCACCAAGGTGGAGGCGATCTTCT<br>TCAACGTGTACGAGGAGACGGTCAAGCTGGTGGTGGCCGTGTTCGAGCGCACCGTCAAGGCGCTGAAGGTG<br>TTCGAGGAGGACTTCAACAAGATCGCCACGAGCGTGTCGGAGCTGTTCCGCACGTTCGCGCAAACGTTCAGC<br>AAGGCCGTCCAGGTGCTGGAGAAGGAGCTGAAGGAGCTGTACAAGCTGGTGCAGGATTACTTCGAC
LacZ	ATCGATAATTTCACCGCCGAAAGGCGCGGTGCCGCTGGCGACCTGCGTTTCACCCTGCCATAAAGAAACTGT<br>TACCCGTAGGTAGTCACGCAACTCGCCGCACATCTGAACTTCAGCCTCCAGTACAGCGCGGCTGAAATCATC<br>ATTAAAGCGAGTGGCAACATGGAAATCGCTGATTTGTGTAGTCGGTTTATGCAGCAACGAGACGTCACGGAAA<br>ATGCCGCTCATCCGCCACATATCCTGATCTTCCAGATAACTGCCGTCACTCCAACGCAGCACCATCACCGCGA<br>GGCGGTTTTCTCCGGCGCGTAAAAATGCGCTCAGGTCAAATTCAGACGGCAAACGACTGTCCTGGCCGTAAC<br>CGACCCAGCGCCCGTTGCACCACAGATGAAACGCCGAGTTAACGCCATCAAAAATAATTCGCGTCTGGCCTT<br>CCTGTAGCCAGCTTTCATCAACATTAAATGTGAGCGAGTAACAACCCGTCGGATTCTCCGTGGGAACAAACGG<br>CGGATTGACCGTAATGGGATAGGTTACGTTGGTGTAGATGGGCGCATCGTAACCGTGCATCTGCCAGTTTGA<br>GGGGACGACGACAGTATCGGCCTCAGGAAGATCGCACTCCAGCCAGCTTTCCGGCACCGCTTCTGGTGCCG<br>GAAACCAGGCAAAGCGCCATTCGCCATTCAGGCTGCGCAACTGTTGGGAAGGGCGATCGGTGCGGGCCTCT<br>TCGCTATTACGCCAGCTGGCGAAAGGGGGATGTGCTGCAAGGCGATTAAGTTGGGTAACGCCAGGGTTTTCC<br>CACTCACGACGTTGTAAAACGACGG

Supplementary Table 4: Primers sequences and concentrations used in this study.

Gene	Forward primer 5' – 3' (conc. used)	Reverse primer 5' – 3'(conc. used)
***A. gambiae* quantitative RT-PCR**		
Lp	CAGCCAGGATGGTGAGCTTAA (300 nM)	CACCAGCACCTTGGCGTT (300 nM)
RPL19	CCAACTCGCGACAAAACATTC (300 nM)	ACCGGCTTCTTGATGATCAGA (900 nM)
***P. berghei* quantitative RT-PCR**		
AP2-sp	AAGAAAAGGCGTCGACATC (50 nM)	ACATCCGTTTGTTCCAACTAA (50 nM)
Slarp	TGCCTTGTGCCGATTC (300 nM)	GGTTTCGTGCTCTTGAGTAGA (50 nM)
CSP	ACCATTTTAGTTGTAGCGTCACTTTTAT (300 nM)	GGGCTTGGATGCTTTTATTTTGT (300 nM)
Puf2	TTATCTTCTCACAAATATGCCTGC (200 nM)	ATTTCCCTCAGGAATGTCGTCAAC (200 nM)
UIS1	GAAAAGTATAAGAATAAGTTTGTTAGTC (200 nM)	GATTTATCCTGAACAATATGAATTCC (200 nM)
UIS4	CCAAACCAAGCGATCATACATACAG (200 nM)	CTTCACCCACTAAATCGCTTAATTC (200 nM)
CDPK6	AAGCATTAAATAAAGCGAATACATCCTTAC (300 nM)	TGCTATGCACAGAAATGAAAA (900 nM)
CytB	GTTTTTAGTGCTAGAGAATATTCAGTTC (900 nM)	TATCTTGTGGTAATTGACATCCTATC (300 nM)
SDH2	GTTATTTTAGGATCAGGATGGGGTG (50 nM)	CACTACATAAACAAGGTAACAAAGGAG (300nM)
18S	AAGCATTAAATAAAGCGAATACATCCTTAC (300 nM)	GGAGATTGGTTTTGACGTTTATGTG (300 nM)

SUPPLEMENTARY METHODS

Quantitative RT-PCR

A. gambiae

Total RNA from 15 mosquitoes was extracted with TriReagent (Sigma) according to the suppliers' instructions. Total RNA (2 μg) was converted to cDNA using the RevertAid H Minus Reverse Transcriptase (Fermentas) and random hexamers (Fermentas). Quantitative PCR reactions were run on a StepOnePlus™ Real-Time PCR instrument (Applied Biosystems) using the Power PCR Master Mix (Applied Biosystems) according to the manufacturer's instructions. Gene encoding ribosomal protein L19 (RPL19) served as an internal control to normalize gene expression. Primers sequences and working concentrations are indicated in Supplementary Table 4 (Rono et al., 2010).

P. berghei

Pellets of 40,000 to 130,000 isolated salivary gland *P. berghei* sporozoites were snap-frozen and kept at -80°C until further processing. RNA was extracted with RNeasy kit (Qiagen) according to the supplier's instructions. Total RNA was DNAse-treated, eluted in 30 μl of elution buffer and converted to cDNA using the RevertAid H Minus Reverse Transcriptase (Fermentas) with a 1:1 mix of random hexamers and oligo dT (Fermentas). Quantitative PCR reactions were run on a StepOnePlus™ Real-Time PCR instrument (Applied Biosystems) using the Power PCR Master Mix (Applied Biosystems). Manufactory chemistry cycling parameters were used for the holding and melt curve stage but a modified double step cycling stage (55°C for 1 min, 60°C for 45 sec) was introduced due to the high AT-content of *Plasmodium* genome. The relative expression level of each gene was normalized to expression levels of the ribosomal RNA gene *18s* (primers' sequences and concentrations in Supplementary Table 4).

Transmission electron microscopy

Infected midguts were dissected in PBS, embedded in low melting agarose (4%) and fixed overnight in 2.5% EM grade glutaraldehyde at 4°C. Midguts were post fixed in 0.5% osmium tetroxide, contrasted with tannic acid and 2% uranyl acetate, dehydrated, and embedded in epoxy resin. After polymerization, sections were cut at 60 nm and contrasted with lead citrate. Specimens were analyzed in a Leo 906E transmission electron microscope at 100KV (Zeiss) using digital camera (Morada).

Scanning electron microscopy

Salivary gland sporozoites (18dpi) were dissected and deposited onto bovine serum albumin-coated glass slides and incubated at 37°C. After fixation with 2% formaldehyde, samples were labelled with a mouse monoclonal antibody against CSP (clone 3D11, BEI Resources, 1.5 μgml^{-1}) and the secondary goat anti-mouse 18 nm gold-conjugated antibody (1:80, Jackson ImmunoResearch, 115-215-146). After labelling, samples were kept in 2.5% glutaraldehyde and then dehydrated in a graded ethanol series, dried in carbon dioxide at critical point and vacuum coated with 3 nm Carbon-Platinum. Imaging was performed using both in-lens and back-scatter signals in a LEO 1550 (Zeiss) scanning-electron microscope at 20 kV acceleration voltage.

SUPPLEMENTARY REFERENCES

Rono, M.K., Whitten, M.M., Oulad-Abdelghani, M., Levashina, E.A., and Marois, E. (2010). The major yolk protein vitellogenin interferes with the anti-*Plasmodium* response in the malaria mosquito *Anopheles gambiae*. PLoS Biol *8*, e1000434.

Shiao, S.H., Whitten, M.M., Zachary, D., Hoffmann, J.A., and Levashina, E.A. (2006). *Fz2* and *cdc42* mediate melanization and actin polymerization but are dispensable for *Plasmodium* killing in the mosquito midgut. PLoS Pathog *2*, e133.
